# Oncogenic RARγ isoforms promote head and neck cancer proliferation through vinexin-β-mediated cell cycle acceleration and autocrine activation of EGFR signal

**DOI:** 10.7150/ijbs.100351

**Published:** 2025-01-01

**Authors:** Yu-Chu Su, Shang-Yin Wu, Keng-Fu Hsu, Shih-Sheng Jiang, Ping-Chung Kuo, Ai-Li Shiau, Chao-Liang Wu, Yang-Kao Wang, Jenn-Ren Hsiao

**Affiliations:** 1 Clinical Medicine Research Center, National Cheng Kung University Hospital, College of Medicine, National Cheng Kung University, 138, Sheng Li Road, Tainan, Taiwan, 70456.; 2 Department of Otolaryngology, National Cheng Kung University Hospital, College of Medicine, National Cheng Kung University, 138, Sheng Li Road, Tainan, Taiwan, 70456.; 3 Department of Oncology, National Cheng Kung University Hospital, College of Medicine, National Cheng Kung University, 138, Sheng Li Road, Tainan, Taiwan, 70456.; 4 Department of Obstetrics and Gynecology, National Cheng Kung University Hospital, College of Medicine, National Cheng Kung University, 138, Sheng Li Road, Tainan, Taiwan, 70456.; 5 National Institute of Cancer Research, National Health Research Institutes, 35, Keyan Road, Zhunan Town, Miaoli County, Taiwan, 350401.; 6 School of Pharmacy, College of Medicine, National Cheng Kung University, 1, University Road, East Dist., Tainan, Taiwan, 70101.; 7 Department of Microbiology and Immunology, College of Medicine, National Cheng Kung University, 1, University Road, East Dist., Tainan, Taiwan, 70101.; 8 Ditmanson Medical Foundation Chia-Yi Christian Hospital, 539, Zhongxiao Rd., East Dist., Chiayi, Taiwan, 60002.; 9 Department of Biochemistry and Molecular Biology, College of Medicine, National Cheng Kung University, 1, University Road, East Dist., Tainan, Taiwan, 70101.; 10 Department of Cell Biology and Anatomy, College of Medicine, National Cheng Kung University, 1, University Road, East District, Tainan, Taiwan, 70101.; 11 Institute of Basic Medical Sciences, College of Medicine, National Cheng Kung University, 1, University Road, East Dist., Tainan, Taiwan, 70101.; 12 Department of Otolaryngology, College of Medicine, National Cheng Kung University, 1, University Road, East Dist., Tainan, Taiwan, 70101.

**Keywords:** Head and neck cancer (HNC), retinoic acid receptor gamma (RARγ), isoform, vinexin-β, epidermal growth factor receptor (EGFR)

## Abstract

Results of retinoid-based therapies in head and neck cancer (HNC) are generally disappointing, indicating a lack of understanding of retinoic acid signaling. The role of retinoic acid receptor gamma (RARγ) and its isoforms in HNC is yet to be established. In this study, we found that RARγ1, 2, 4 are the predominant RARγ isoforms expressed in various types of human cancers, including HNC. The mechanistic study revealed that RARγ1, 2, 4 enhanced the proliferation of HNC cells by accelerating cell cycle progression through interaction with vinexin-β, as well as by ligand-dependent activation of EGFR with downstream Akt, ERK, Src, and YAP signaling pathways. Retinoic acid binding and CDK7-dependent phosphorylation on specific serine residue at the AF-1 domain are mandatory for RARγ-mediated growth promotion of HNC. Knockdown of RARγ abolished proliferation of cultured HNC cells, and completely prevented tumor growth in xenografted nude mice. Similar effects were observed in various human cancer types other than HNC. Our results indicate that RARγ-targeting approach could be a promising therapeutic and chemopreventive strategy for human cancers.

## Introduction

Head and neck cancer (HNC) is the seventh most common type of cancer worldwide, with more than 90% of HNCs being squamous cell carcinomas (HNSCC). While the 5-year overall survival rates of early-stage (stage I, II) patients are around 70-90%, about half of the advanced-stage (stage III, IV) patients still fail treatment despite appropriate therapy 1. In addition, more than 10% of HNC patients who survive their primary treatment will develop second primary cancer 2, further compromising the long-term survival of these patients.

Retinoic acids (RA) are active metabolites of vitamin A (retinol) that modulate important cellular functions, including proliferation, differentiation, and survival. The pleiotropic functions of RA are mainly conducted by binding to its receptors, including retinoic acid receptors (RARs) and retinoic X receptors (RXRs). Ligand-bound RARs/RXRs act as transcriptional factors to regulate the expression of RA-responsive genes, known as RA's genomic effects. In addition to the genomic effects, RA can directly activate cellular kinase cascades to covey nongenomic effects 3,4. Hence, dysregulation of RA signaling has long been implicated in oncogenesis of human cancers 5-7. However, although retinoids (derivatives from vitamin A, including RA) have long been used in chemotherapeutic or chemopreventive trials for HNC, results from these studies are generally disappointing 8, indicating that a knowledge gap does exist in our understanding of retinoic acid signaling.

There are three main subtypes of human RARs, namely, RARα, RARβ, and RARγ, with several isoforms noted for each RAR subtype 6. Among these RARs, RARβ2 is well recognized as a tumor suppressor gene inactivated by hypoacetylation or promoter hypermethylation in more than 70% of cancers 7. Although RARα was initially shown to be an oncogenic driver as a fusion product of promyelocytic leukemia (*PML*) and *RARA* genes in acute promyelocytic leukemia (APL) 9, recent reports have demonstrated that native RARα possesses oncogenic properties itself 10-14. In contrast to RARβ2 and RARα, the role of RARγ in human cancers is yet to be defined. For instance, while RARγ is considered an oncogene in some human cancers 15, contradictory reports also demonstrated that RARγ could be a tumor suppressor gene rather than an oncogene 16,17. It remains unclear whether these inconsistent observations result from the differential expression of RARγ isoform(s) across tumor types or from tumor-specific effects of RARγ.

This study aims to decipher the role of RARγ isoforms in carcinogenesis and progression of HNC, and to explore the feasibility of RARγ-targeting approach as a therapeutic and/or chemo-preventive strategy for HNC and other types of human cancers. Our results demonstrated that RARγ isoform 1, 2, 4 are the predominant RARγ isoforms expressed in various types of human cancer cells, including HNC. Mechanistically, RARγ1, 2, 4 enhanced the proliferation of HNC cells by vinexin-mediated cell cycle acceleration, and by autocrine activation of EGFR, which subsequently triggers downstream ERK, Akt, and YAP signaling pathways. Both RA-binding and phosphorylation of regulatory serine residue at the AF-1 domain are required for the growth-promoting effect of RARγ. Knockdown of RARγ abolished proliferation of HNC cells *in vitro*, and effectively prevented tumor growth *in vivo*. Knockdown of RARγ similarly prevented *in vitro* and *in vivo* tumor growth in various types of human cancer cells other than HNC. Taken together, our results indicate that RARγ-targeting approach could be a promising therapeutic and chemo-preventive strategy for HNC and other types of human cancers.

## Results

### RARγ isoforms 1, 2, 4 are the major RARγ isoforms expressed in HNC and various types of human cancers

**Figure 1:**
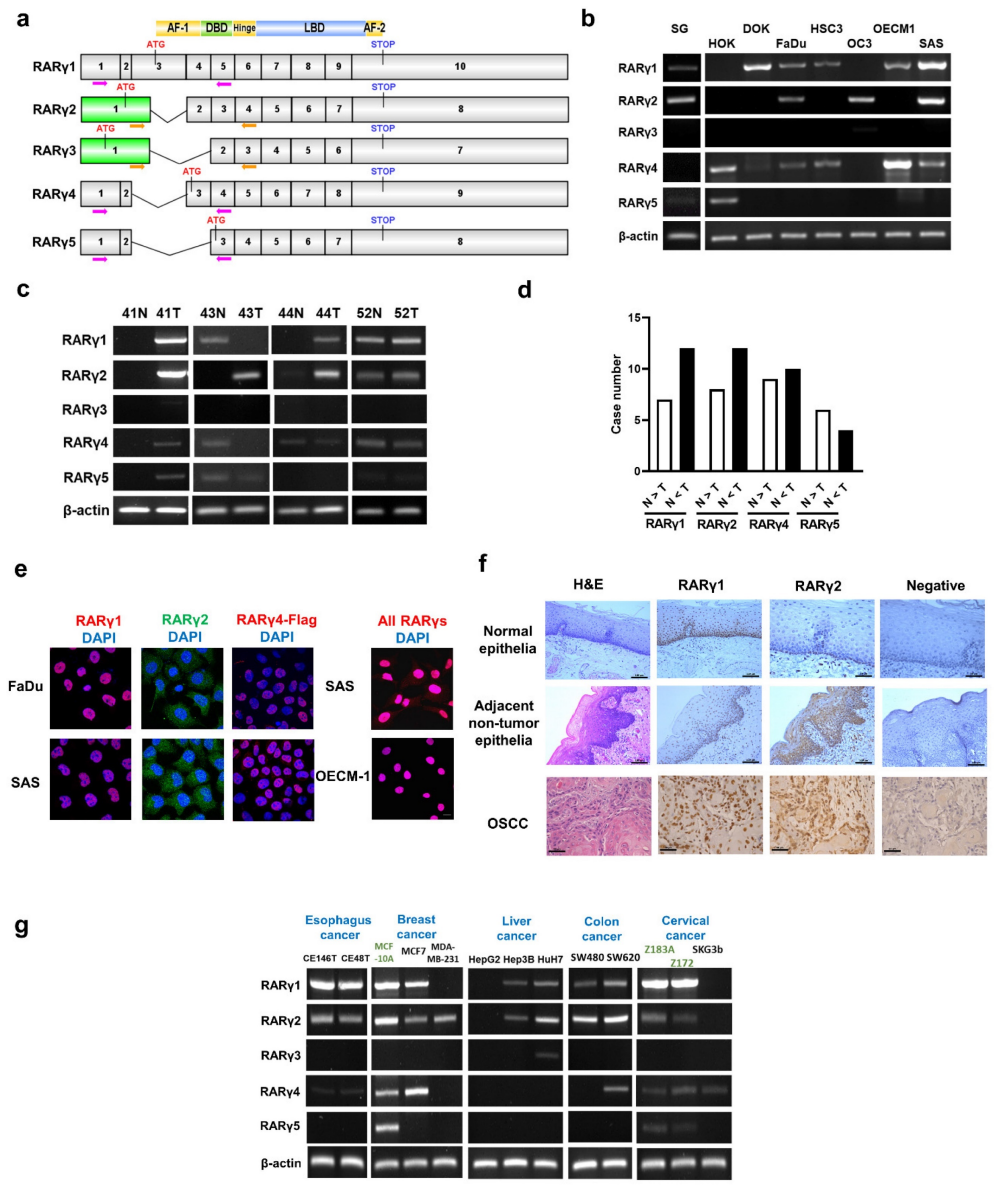
**Expression of RARγ isoforms in head and neck cancer (HNC) tissues and various cell lines. (a)** Schematic illustration showing the cDNA structure of the five RARγ isoforms. Arrows indicate primer pairs designed to detect the expression of each RARγ isoform. **(b)** The mRNA expression of RARγ isoforms in HOK (primary human normal oral keratinocytes), SG (immortalized human oral keratinocytes), DOK (dysplstic human oral keratinocytes) and various HNC cell lines (FaDu, HSC3, OC3, OECM1, SAS). **(c)** Representative RT-PCR results of RARγ isoform detection in oral squamous carcinomas (OSCC) (T) and their corresponding adjacent non-tumor epithelia (N). **(d)** The relative abundance of each RARγ isoform in the 20 pairs of OSCC (T) and their adjacent non-tumor epithelia (N). **(e)** Confocal microscopic study to reveal the localization of RARγ1, RARγ2, flag-tagged RARγ4 and all RARγs in HNC cells (RARγ1, RARγ4-flag and all RARγs in red fluorescence; RARγ2 in green fluorescence; Nuclei were stained blue with DAPI). Scale bar: 10 μm.** (f)** Representative immunohistochemical (IHC) study conducted on expression and localization of RAR**γ**1, RAR**γ**2 in normal epithelia (upper panel), adjacent non-tumor epithelia of oral squamous cell carcinoma (OSCC) (middle panel), and OSCC tumors (lower panel). Scale bar: 100 μm (upper and middle panel); 50 μm (lower panel) **(g)** Expression patterns of RARγ isoforms in various types of human cancer, and immortalized non-cancer cell lines (MCF10A, Z183A, and Z172).

Figure 1a illustrates the mRNA structures of the five major isoforms of RARγ (RARγ 1-5). Figure 1b demonstrates the expression patterns of the five RARγ isoforms in normal human oral keratinocytes (HOK), immortalized oral keratinocytes (SG), dysplastic oral keratinocytes (DOK), and head and neck cancer cell lines (FaDu, HSC3, OC3, OECM1, SAS). It was clearly shown that the RARγ1, 2, 4 were the predominant RARγ isoforms expressed in immortalized SG, DOK, and head and neck cancer cells. In contrast, only normal primary human oral keratinocytes (HOK) expressed RARγ5. RARγ3 is not expressed in any of these cells. Using 20 pairs of fresh frozen OSCCs and their corresponding adjacent non-tumor epithelia, we confirmed that RARγ1, 2, 4 were the main RARγ isoforms expressed in tumor tissues (Fig. 1c). RARγ5 was either not expressed, or expressed in a relatively low level in OSCC specimens. In more than half of the paired specimens, the expression levels of RARγ1, 2, 4 were higher in tumor tissue compared to their corresponding non-tumor epithelia (Fig. 1d).

The cellular localization of RARγ1, 2, 4 was further studied using confocal microscopy. Under normal culture conditions, native RARγ1 and the exogenous flag-tagged RARγ4 were strictly localized in the nuclei of FaDu and SAS cells, while native RARγ2 was found in both cytoplasm and the nucleus (Fig. 1e). Immunohistochemical study was also performed to study the expression patterns of RARγ isoforms in formalin-fixed, paraffin-embedded tissues from normal oropharyngeal epithelia (n=10), non-tumor epithelia adjacent to OSCC (n=10) and OSCCs (n=10). Consistent with *in vitro* findings, RARγ1 was detected exclusively in the nuclei of normal epithelial cells (Fig. 1f, upper panel), adjacent non-tumor epithelial cells of OSCC (Fig. 1f, middle panel), and tumor cells (Fig. 1f, lower panel). In contrast, RARγ2 was located in both the cytoplasm and nucleus. Notably, in normal oropharyngeal epithelia, RARγ2 was only weakly expressed and confined to the basal epithelial cell layer. In contrast, RARγ2 was expressed at a higher level in the non-tumor epithelial cells adjacent to OSCC and diffusely expressed in multiple epithelial layers. In OSCCs, RARγ2 was predominantly expressed in the invasion front of tumor islands. These results indicated that differential expression of RARγ isoforms occurs early in the carcinogenesis of HNC.

We further investigated the expression patterns of RARγ isoforms in various types of human cancer cells. As shown in Fig. 1g, similar to the findings in HNC, RARγ1, 2, 4 were the predominant isoforms expressed in esophageal (CE146T and CE48T), breast (MCF7 and MDA-MB-231), liver (Hep3B and HuH7), colon (SW480 and SW620), and cervical (SKG3b) cancer cells. In contrast, RARγ5 was expressed only in the non-cancer breast epithelial (MCF10A) and cervical epithelial (Z183A and Z172) cell lines, suggesting that this shift in RARγ isoform expression is a common feature of human cancers.

### RARγ1, 2, 4 enhance proliferation in immortalized oral keratinocytes and HNC cells

**Figure 2:**
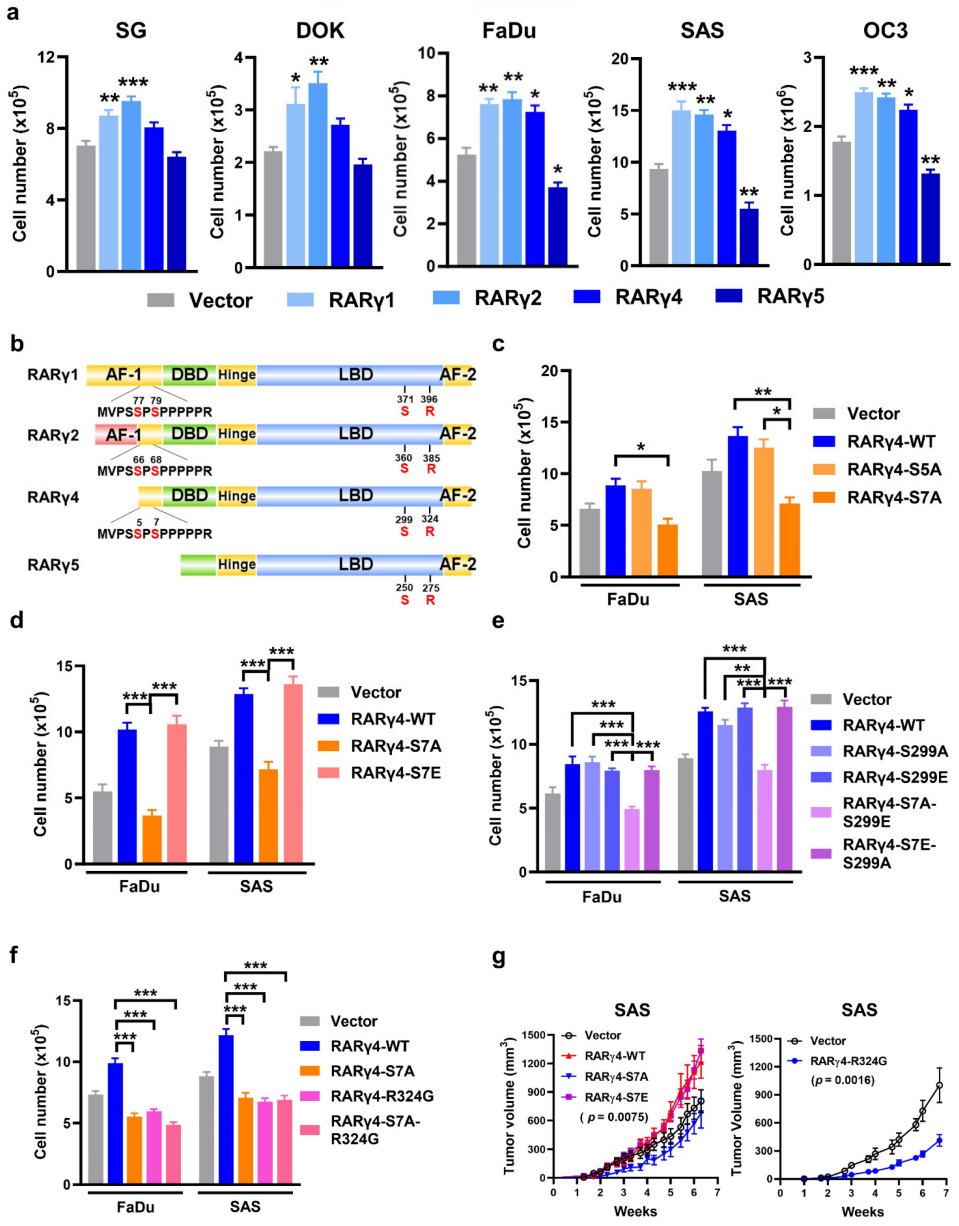
** Role of RARγ isoforms in growth-modulation of HNC cells. (a)** Proliferation assays showing the growth-modulation effects of RAR**γ** isoforms in SG, DOK, FaDu, SAS and OC3 cells. **(b)** Schematic illustration showing the protein structures of RAR**γ**1, 2, 4, 5. Four distinct domain structures are noted in RAR**γ**, including an AF-1 domain, a DNA-bindin domain (DBD), a ligand-binding domain (LBD), and a C-terminal AF-2 domain. The AF-1 domain of RAR**γ**1, 2, 4 contains a proline-rich area with two phospho-regulatory serine residues. Another phospho-regulatory serine residue is located at the LBD (Ser^299^ of RAR**γ**4).** (c)** Phospho-defective Ser^7^ (RARγ4-S7A) suppressed RAR**γ**4-mediated growth-promotion of FaDu and SAS cells. **(d)** The effect of phospho-mimic Ser^7^ (RAR**γ**4-S7E) on HNC cell proliferation. **(e)** The phosphorylation status of Ser^299^ did not impact RAR**γ**4-mediated proliferation of FaDu and SAS cells. **(f)** RAR**γ**4-enhanced HNC proliferation is RA-dependent. Mutation of the RA-binding pocket (RAR**γ**4-R324G) significantly impaired RA binding and attenuated RAR**γ**4-mediated growth promotion in FaDu and SAS cells.** (g)** Nude mice inoculated subcutaneously with SAS-RAR**γ**4, SAS-RAR**γ**4-S7A, SAS-RAR**γ**4-R324G, or vector control (n = 8). Tumor volumes were measured twice a week. *p < 0.05; **p < 0.01; ***p < 0.001.

We next investigated the role of RARγ isoforms in HNC carcinogenesis. The protein levels of overexpressed, Flag-tagged RARγ4 and its mutants (S7A, S299A, R324G) were shown in Supplemental Fig. S1a. The flag-tagged RARγ isoforms were overexpressed in SAS cells, as illustrated in Fig. S1b. As shown in Figure 2a, overexpression of RARγ1, 2, 4 significantly enhanced the proliferation of immortalized (SG), dysplastic (DOK), and HNC cells (FaDu, SAS, and OC3). In contrast, the forced expression of RARγ5 attenuated the growth of FaDu, SAS, and OC3 cells. Figure 2b depicts the protein structures of RARγ isoforms, highlighting four main structure domains: The N-terminal AF-1 domain (AF-1), the DNA binding domain (DBD), the ligand-binding domain (LBD) for RA binding, and the C-terminal AF-2 domain 18. Although RARγ1 and RARγ2 have a unique and longer AF-1 domain compared to RARγ4, a common proline-rich area is present in RARγ1, 2, 4. In contrast, RARγ5 lacks the AF-1 domain with a short truncation of the DNA binding domain. It was previously reported that the two serine residues on the proline-rich area of the AF-1 domain (Ser^77, 79^ of RARγ1 and Ser^66, 68^ of RARγ2) were functionally relevant and could be phospho-regulated by CDK7 19 or p38 20, respectively. We then hypothesized that the growth-modulating effects of RARγ isoforms could be attributed to the phosphorylation of these serine residues. Considering that RARγ4 was the shortest RARγ isoform that could enhance the proliferation of HNC cells (Fig. 2a), we used RARγ4 as a model molecule to study the growth-promoting effect of RARγ. Fig. 2c demonstrated that mutation of Ser^5^ into a phospho-defective alanine (RARγ4-S5A) had no impact on RARγ4-mediated growth-promotion of HNC cells. In contrast, forced expression of a phospho-defective RARγ4 mutant at Ser^7^ (RARγ4-S7A) significantly attenuated the growth of FaDu and SAS cells. Mutation of Ser^7^ residue into a phospho-mimetic glutamic acid (RARγ4-S7E) did not further enhance the proliferation of HNC cells (Fig. 2d). In a proposed model of RARα activation, Ser^369^ phosphorylation (Ser^299^ in RARγ4) by p38MAPK/MSK1 was essential for coordinate phosphorylation of Ser^77^ (Ser^7^ in RARγ4) 21. To test this possibility, phospho-defective (RAR4-S299A), phospho-mimetic (RARγ4-S299E) and double mutated (RAR4-S7A-S299E and RAR4-S7E-S299A) RARγ4 mutants were constructed. However, forced expression of the phospho-defective mutant on Ser^299^ of RARγ4 (RAR4-S299A) had no impact on the growth-promoting effect of RARγ4 (Fig. 2e). Among all these constructs, only RARγ4 mutant containing the S7A mutation (RARγ4-S7A-S299E) attenuated growth of HNC cells (Fig. 2e), indicating that the Ser^299^ phosphorylation is dispensable in controlling Ser^7^ phosphorylation and RARγ4-mediated growth-promotion of HNC cells.

We next determined whether the RARγ4-mediated pro-proliferative effect of HNC cells requires ligand (RA) binding. Mutation of an arginine residue (Arg^394^, corresponding to Arg^324^ in RARγ4) within the LBD of RARα has been shown to completely abolish the association between RA and RARα 22. Since the LBD was conserved among all subtypes of RAR, a ligand-binding defective RARγ4 mutant was similarly constructed (RARγ4-R324G, Fig S1a). Forced expression of RARγ4-R324G resulted in a comparable growth inhibition of HNC cells similar to RARγ4-S7A (Fig. 2f), indicating that the growth-promoting effect of RARγ4 is also RA-dependent. The above *in vitro* findings were further confirmed *in vivo* using a xenograft model with SAS-transplanted nude mice (Fig. 2g).

### Interaction between RARγ and vinexin-β serves as a novel cell cycle regulatory mechanism

**Figure 3:**
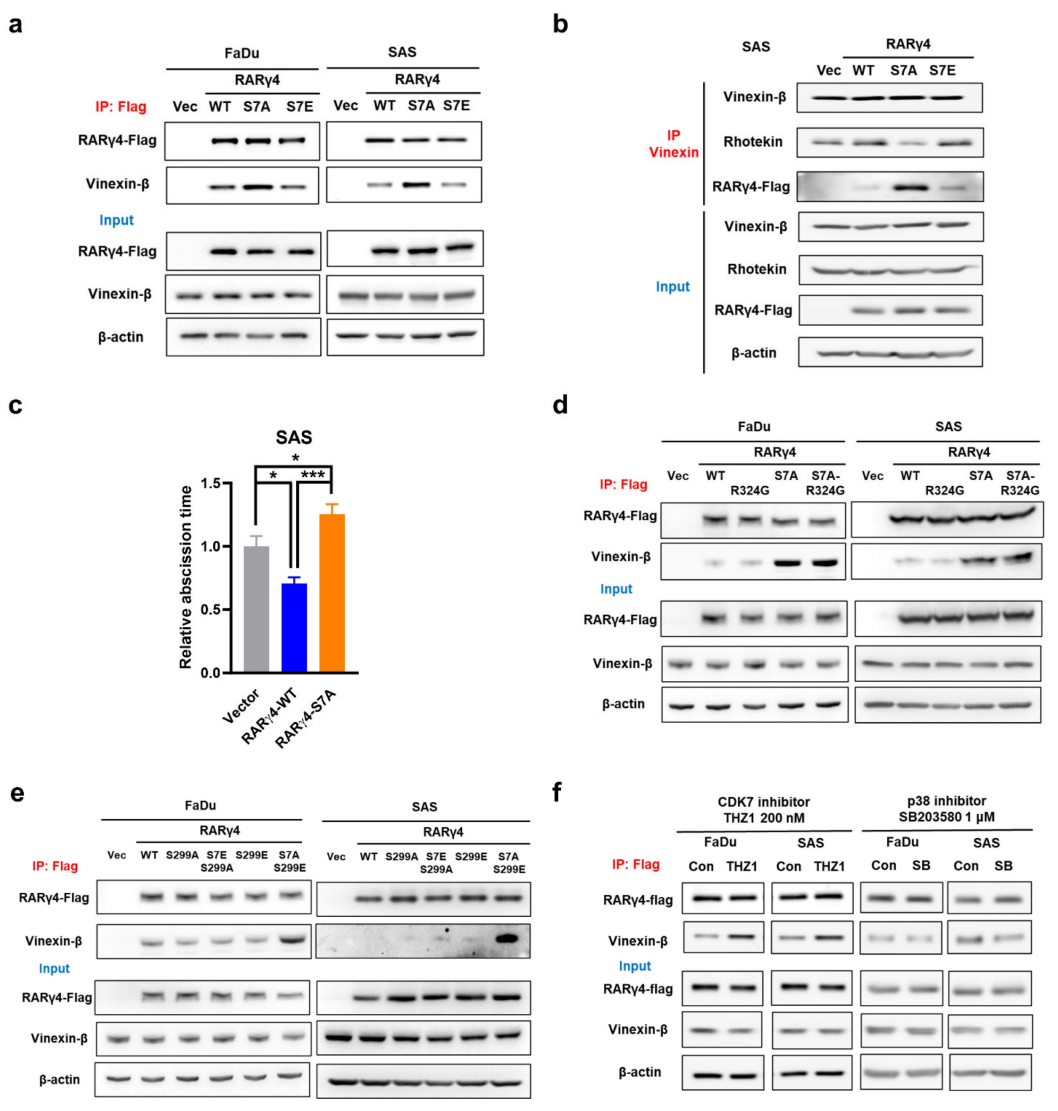
** Interaction of RARγ4 AF-1 domain with vinexin-β as a novel mechanism to regulate M2 abscission in HNC cells. (a)** Phospho-defective RAR**γ**4 (RAR**γ**4-S7A) bound vinexin-β. Cell lysates were immunoprecipitated by an anti-flag antibody, followed by immunoblotting to detect the amount of vinexin-β in each immunocomplex. **(b)** Phospho-defective RAR**γ**4 (RAR**γ**4-S7A) bound vinexin-β and decreased interaction between vinexin-β and rhotekin. Cell lysates were immunoprecipitated by anti-vinexin antibody, followed by immunoblotting to detect the amount of rhotekin and RAR**γ**4 in the immunocomplex. **(c)** SAS cells expressing RAR**γ**4, RAR**γ**4-S7A, and vector control were monitored with time-lapse microscopy to measure the abscission time (*p < 0.05; ***p < 0.001). **(d)** Interaction of AF-1 domain with vinexin-β is ligand-independent. The RA-binding defective RAR**γ**4-R324G did not impact the association of RAR**γ**4 and vinexin-β. **(e)** The phosphorylation status of S299 does not affect the interaction between RAR**γ**4 and vinexin-β. Phospho-defective RAR**γ**4-S299A or phospho-mimetic RAR**γ**4-S299E has no impact on the association between RAR**γ**4 and vinexin-β. **(f)** CDK7, but not p38, mediates phosphorylation of Ser^7^ and modulates the interaction between RAR**γ**4 and vinexin-β. Cells were treated either with CDK7 inhibitor (THZ1, 200 nM) or p38 inhibitor (SB203580, 1 μM) for 6 h and harvested for immunoprecipitation using an anti-flag antibody to examine the interaction between RAR**γ**4 and vinexin-β.

We next aimed to decipher the growth-regulatory mechanism(s) from RARγ isoforms. It was previously shown that the non-phosphorylated AF-1 domain on RARγ1 could associate with vinexin-β. Phosphorylation of Ser^77^ and Ser^79^ on AF-1 domain prevented such binding, leading to dissociation of vinexin-β from RARγ1 18. Notably, a recent report demonstrated that in M2 phase of cell cycle, midbody localization of vinexin-β could recruit rhotekin to facilitate abscission and cell cycle progression 23. We thus hypothesized that the interaction between RARγ AF-1 domain and vinexin-β might be a mechanism for RARγ to regulate cell cycle progression in HNC cells. Figure 3a showed that, in FaDu and SAS expressing wild-type (RARγ4-WT) or phospho-mimetic (RARγ4-S7E) RARγ4, immunoprecipitation of RARγ4 co-precipitated a similar amount of vinexin-β. However, a higher amount of vinexin-β was co-immunoprecipitated in FaDu and SAS cells expressing RARγ4-S7A construct, confirming the association between vinexin-β and the non-phosphorylated AF-1 domain of RARγ4. Meanwhile, immunoprecipitation of vinexin-β in SAS cells expressing RARγ4-S7A co-precipitated a lesser amount of rhotekin, implying a reduced association between vinexin-β and rhotekin (Fig. 3b). We also confirmed that the abscission time was significantly reduced in SAS cells expressing RARγ4-WT, and markedly prolonged in SAS cells expressing RARγ4-S7A (Fig. 3c). Taken together, we confirmed that such RARγ/vinexin-β interaction is a novel cell cycle regulatory mechanism for RARγ-mediated growth promotion of HNC cells.

Given that both Ser^7^ phosphorylation (Fig. 2d) and RA-binding (Fig. 2e) are essential for modulating the RARγ4-mediated growth promotion of HNC, we investigated whether these factors are correlated. Figures 3d and 3e demonstrated that the association of RARγ4 with vinexin-β was not affected in FaDu and SAS cells expressing either RARγ4-R324G or RARγ4-S299A, indicating that the association between RARγ4 AF-1 domain and vinexin-β was independent of RA binding or Ser^299^ phosphorylation. Previous reports have demonstrated that the two serine residues on the AF-1 domain of RARγ could be phosphorylated by either CDK7 19 or p38 20, respectively. However, which kinase is responsible for controlling RARγ4 Ser^7^ phosphorylation and HNC proliferation is unclear. Figure 3f showed that inhibition of CDK7 (with THZ1), rather than inhibition of p38 (with SB203580), enhanced the association between RARγ4 and vinexin-β, indicating that CDK7 is the major cellular kinase to phosphorylate Ser^7^ at AF-1 domain of RARγ4.

**Figure 4:**
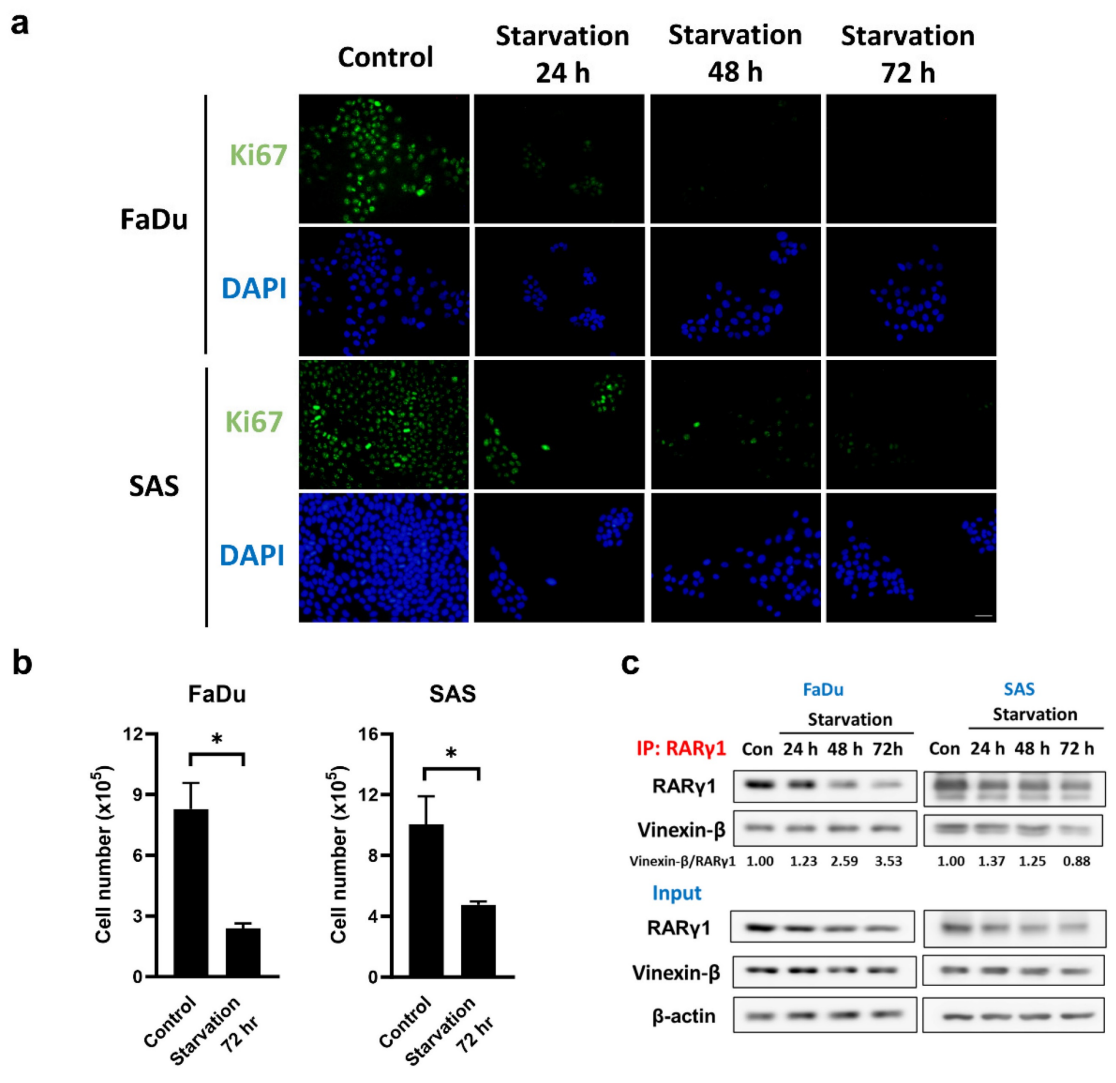
** Serum starvation enhanced the association between RARγ1 and vinexin-β. (a)** The expression of Ki67 was detected in serum-starved FaDu and SAS cells using immunofluorescence staining. Scale bar: 25 μm. **(b)** Total cell numbers were counted after the cells were serum-starved for 72 hours (*p < 0.05). **(c)** The interaction between native RARγ1 and vinexin-β increased under serum starvation conditions. Cell lysates were immunoprecipitated with anti-RARγ1 antibody, followed by immunoblotting to detect the amount of vinexin-β in each immunocomplex.

To eliminate the possibility that the interaction between RARγ4 and vinexin-β, which mediates the cell cycle regulation, represents a non-physiological association due to RARγ4 overexpression, we further examined the native interaction between RARγ1 and vinexin-β in FaDu and SAS cells under serum starved conditions. Figure 4 confirmed that serum starvation did inhibit cell cycle progression and proliferation in FaDu and SAS cells, as indicated by the decreased Ki-67 expression (Fig. 4a) and cell number (Fig. 4b). Using an RARγ1-specific antibody, we demonstrated that vinexin-β could be co-immunoprecipitated with native RARγ1 in both FaDu and SAS cells (Fig. 4c), implying an interaction between these two molecules. Compared to normal proliferating cells (control group without serum starvation), although the amount of RARγ1 in FaDu and SAS cells decreased following serum starvation, the amount of vinexin-β co-immunoprecipitated with RARγ1 remained largely unchanged (except for SAS cells at 72 h) (Fig. 4c), indicating an enhanced association between RARγ1 and vinexin-β (as reflected by the vinexin-β/RARγ1 ratios in Fig. 4c) in cells without active proliferation. These observations were similar to our experimental results conducted on RARγ4 (Fig. 3) and supported a physiological role of RARγ-vinexin-β interaction in cell cycle regulation.

### Oncogenic RARγ isoforms promote HNC proliferation through autocrine activation of EGFR and downstream Akt, ERK, Src, and YAP signaling

**Figure 5:**
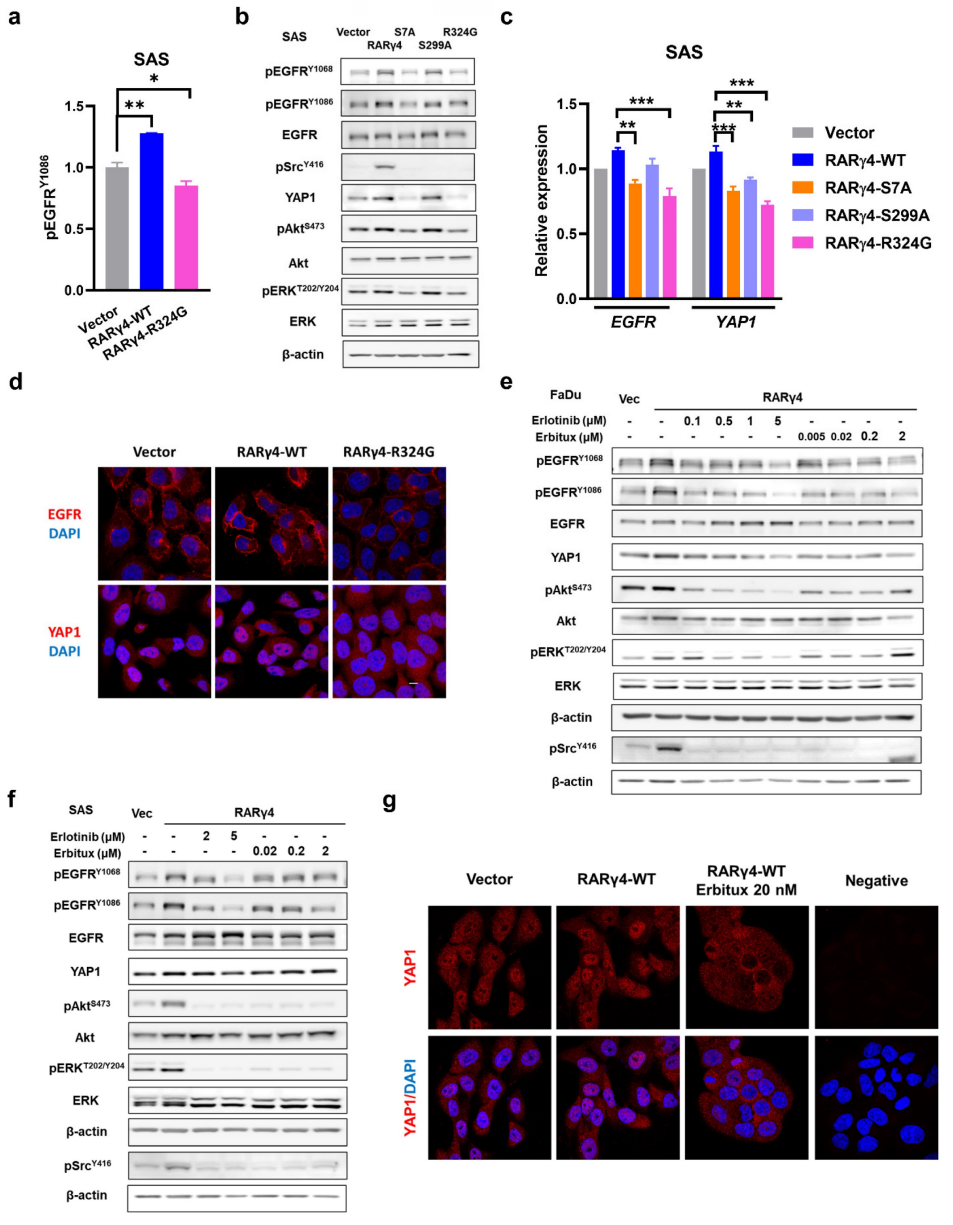
** Activation of EGFR and downstream signaling pathways as novel regulatory mechanisms for RARγ-mediated growth-promotion of HNC cells. (a)** Quantification results from the kinase array study revealed that overexpression of RAR**γ**4 enhanced EGFR phosphorylation. **(b)** In addition to EGFR activation, Src, Akt, and ERK pathways were also activated in SAS cells overexpressing RARγ4. Activation of EGFR, Akt, and ERK were suppressed in SAS cells expressing RAR**γ**4-S7A and RAR**γ**4-R324G. Activation of Src was suppressed in SAS cells expressing RAR**γ**4-S7A, RAR**γ**4-R324G, and RAR**γ**4-S299A mutants. **(c)** Transcriptional regulation of *EGFR* and *YAP1* in SAS cells expressing RARγ4. Upregulation of *EGFR* and *YAP1* were suppressed in SAS cells expressing RAR**γ**4-S7A, RAR**γ**4-R324G, and RAR**γ**4-S299A mutants. (*p < 0.05; **p < 0.01; ***p < 0.001). **(d)** Confocal microscopic study showing that overexpression of RAR**γ**4 in SAS cells increased the protein levels of EGFR and YAP1, and enhanced membrane localization of EGFR and nuclear translocation of YAP1. Activation of EGFR and YAP1 pathways were repressed in SAS cells expressing the RA-binding defective mutant RAR**γ**4-R324G. Scale bar: 10 μm.** (e and f)** Inhibition of EGFR signaling repressed activation of downstream Src, Akt, and ERK signaling pathways in FaDu and SAS cells. FaDu **(e)** and SAS **(f)** cells expressing RAR**γ**4 were treated with various concentrations of either Erlotinib (a kinase inhibitor of EGFR) or Erbitux (a monoclonal antibody against EGFR) for 24 h. **(g)** Activation of YAP1 in RAR**γ**4 expressing SAS cells was suppressed by the administration of Erbitux. Anti-EGFR treatment translocated YAP1 from the nucleus to cytoplasm in SAS-RAR**γ**4 cells. Scale bar: 10 μm.

We have demonstrated that both AF-1 domain phosphorylation and RA binding were required for the RARγ4-mediated proliferation of HNC cells (Fig. 2c-g). However, considering that (1) AF-1 domain phosphorylation is independent of RA binding (Fig. 3d), and (2) the growth-inhibitory effect of HNC cells expressing a double mutated RARγ4 (RARγ4-S7A-R324G) did not significantly differ from HNC cells expressing a single mutated construct (RARγ4-S7A or RARγ4-R324G) (Fig. 2f), we thus speculated that the growth-modulating effect of AF-1 domain and ligand domain could converge on a specific signaling pathway. An array-based study was hence conducted to explore potential RARγ4-modulated signaling pathways (Supplementary Table S1). We noticed that the phosphorylation of EGFR was markedly activated in SAS cells expressing RARγ4-WT, and was inversely repressed in SAS cells expressing RARγ4-R324G (Fig. 5a, 5b, and Table S1). Confirmatory western blot experiments demonstrated that in addition to EGFR activation, Akt, ERK, and Src pathways were also activated in SAS cells expressing RARγ4-WT (Fig. 5b). Conversely, expression of RARγ4-S7A or RARγ4-R324G in SAS cells repressed activation of the above pathways (Fig. 5b). Compare to vector control, the protein levels of phosphor-EGFR and YAP1 were both increased in SAS cells expressing wide-type RARγ4, and were decreased in SAS cells expressing RARγ4-S7A or RARγ4-R324G (Fig. 5b). Quantitative RT-PCR study confirmed that overexpression of RARγ4 induced a non-significant increase of *EGFR* and *YAP1* expression in SAS cells, while defective mutants of RARγ4 (RARγ4-S7A, -S299A, -R324G) suppressed the transcriptional levels of *EGFR* and *YAP1* (Fig. 5c).

A recent study demonstrated that EGFR signaling activation enhanced YAP1 nuclear translocation, leading to the upregulation of growth-promoting genes 24. We therefore investigated whether RARγ4 could also modulate YAP1 functionally. As expected, RARγ4 expression in SAS cells promoted EGFR activation and YAP1 nuclear localization, indicating a functional activation of both molecules (Fig. 5d). In contrast, expression of RARγ4-R324G mutant in SAS cells inhibited EGFR activation and caused YAP1 cytoplasmic retention, confirming that RARγ4 regulates YAP1 function.

We next sought to determine whether Akt, ERK, Src, and YAP1 activation could be coordinated by EGFR activation. Administration of an EGFR kinase inhibitor (Erlotinib), or an EGFR blocking antibody (Erbitux) to RARγ4-expressing FaDu or SAS cells simultaneously repressed activation of EGFR, Akt, ERK, and Src pathways (Fig. 5e and 5f), implying that Akt, ERK, and Src are downstream signaling pathways coordinated by EGFR activation. Administration of Erbitux induced YAP1 cytoplasmic retention (Fig. 5g), implying that the YAP pathway is also modulated by RARγ4-mediated EGFR signaling. It was reported that EGFR activation could be ligand-dependent or ligand-independent 25. The successful co-inhibition of EGFR, Akt, ERK, Src, and YAP activation by the monoclonal antibody against the extracellular domain of EGFR (Erbitux) indicates that the RAR4-mediated EGFR activation is ligand-dependent.

**Figure 6:**
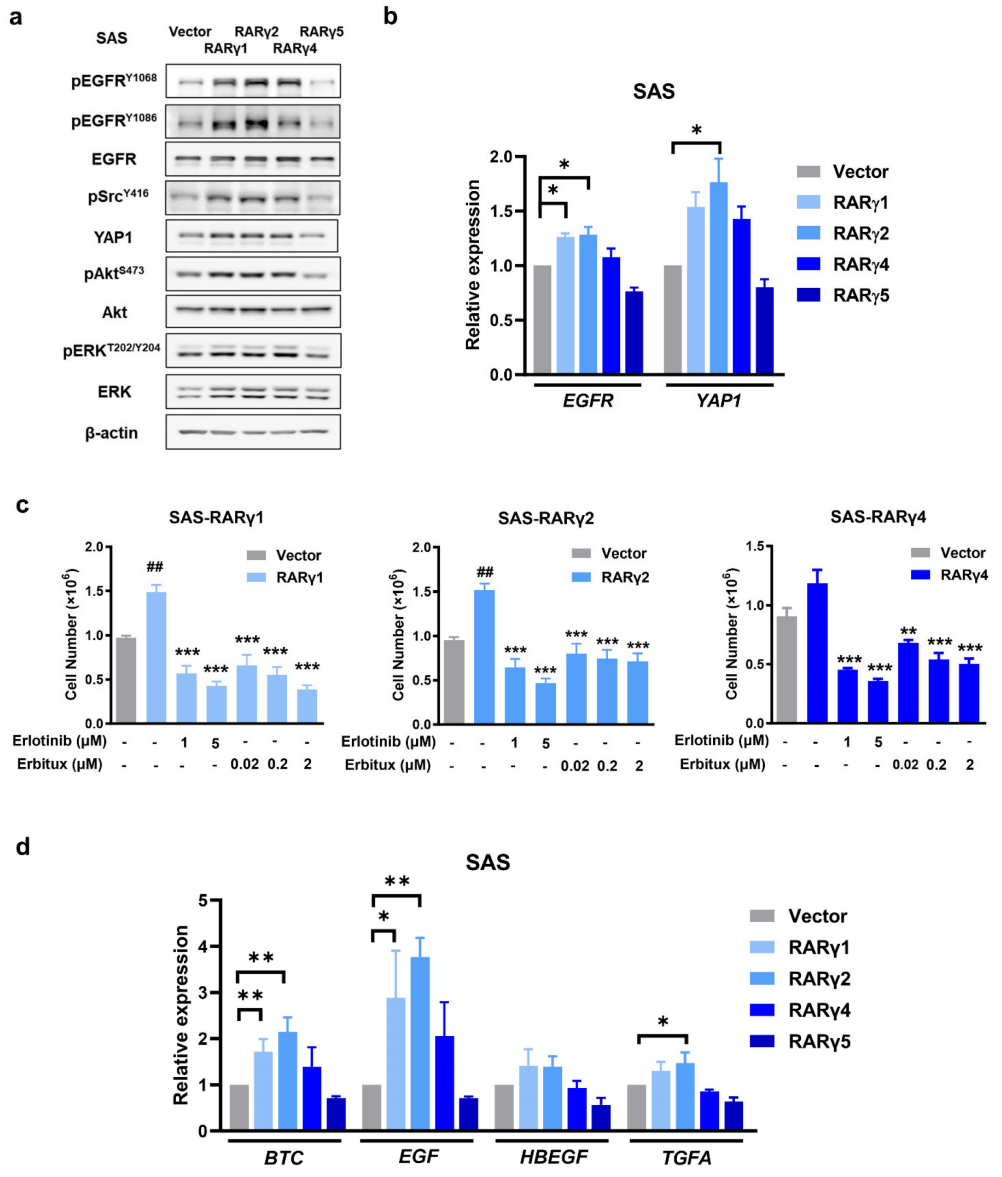
** RAR**γ** modulated ligand-dependent activation of EGFR to enhance proliferation of HNC cells. (a)** In SAS cells, overexpression of RAR**γ**1, 2, 4 activated EGFR, Akt, ERK, Src and increased protein levels of EGFR and YAP1. The activation of EGFR-related signaling pathways was suppressed in SAS cells expressing RAR**γ**5. **(b)** Transcriptional up-regulation of *EGFR* and *YAP1* by RAR**γ**1, 2, 4 but not RAR**γ**5. **(c)** Inhibition of EGFR activation suppressed RAR**γ**1, 2, 4-mediated growth-promotion of SAS cells. Cells expressing RAR**γ**1, 2, 4 were treated with either Erlotinib or Erbitux. Cell numbers were calculated in each group on day 7. **(d)** Expression of EGFR ligands were detected in SAS cells expressing RAR**γ**1, 2, 4. (#, vector vs RAR**γ**1, 2. *, RAR**γ**1, 2, 4 vs EGFR inhibitor treated group. *p < 0.05; **p < 0.01; ***p < 0.001).

Similar studies were conducted in SAS cells expressing each RARγ isoform, demonstrating that these regulatory mechanisms are not unique to RARγ4. Compared to the vector control, the expression of RARγ1, 2, 4 consistently activated EGFR, Akt, ERK, Src, and YAP pathways, whereas RARγ5 expression suppressed the activation of these pathways (Fig. 6a). The transcriptional levels of *EGFR* and *YAP1* were consistently up-regulated in SAS (Fig. 6b) and FaDu (Supplementary Fig. S2) cells expressing RARγ1, 2, 4, and were inversely down-regulated in SAS and FaDu cells expressing RARγ5.

To ensure that RARγ-mediated EGFR activation did modulate HNC proliferation, FaDu or SAS cells expressing each RARγ isoform were treated with either Erlotinib or Erbitux (Fig. 6c and Supplementary Fig. S3). As expected, both Erlotinib and Erbitux treatment suppressed the growth-promoting effect mediated by RARγ1, 2, 4 in FaDu and SAS cells, implying that RARγ 1, 2, 4 enhanced the growth of HNC cells through activation of EGFR signaling.

Since RARγ-mediated EGFR activation is ligand-dependent (Fig. 5e and 5f), we hypothesize that RARγ may simultaneously upregulate its ligand(s) to enhance HNC proliferation through autocrine signaling. To test this hypothesis, we examined the expression levels of high-affinity EGFR ligands, including betacellulin (*BTC*), epidermal growth factor (*EGF*), heparin-binding epidermal growth factor-like growth factor (*HB-EGF*), and transforming growth factor-α (*TGFA*) 26 in SAS cells expressing different RARγ isoforms. Figure 6d shows that the transcriptional levels of *BTC* and *EGF* were significantly upregulated by RARγ1 and RARγ2 in SAS cells, while RARγ4 induced a modest, non-significant increase in *BTC* and *EGF*. The *TGFA* expression was also upregulated by RARγ2 in SAS cells. These findings were further confirmed in FaDu cells (Supplementary Fig. S4). Taken together, our results demonstrate that the RARγ1, 2, 4-mediated growth promotion of HNC cells primarily occurs through autocrine, ligand-dependent activation of EGFR and the coordination of downstream signaling pathways.

### RARγ-targeting as a therapeutic/chemopreventive strategy for the treatment of human cancers

**Figure 7:**
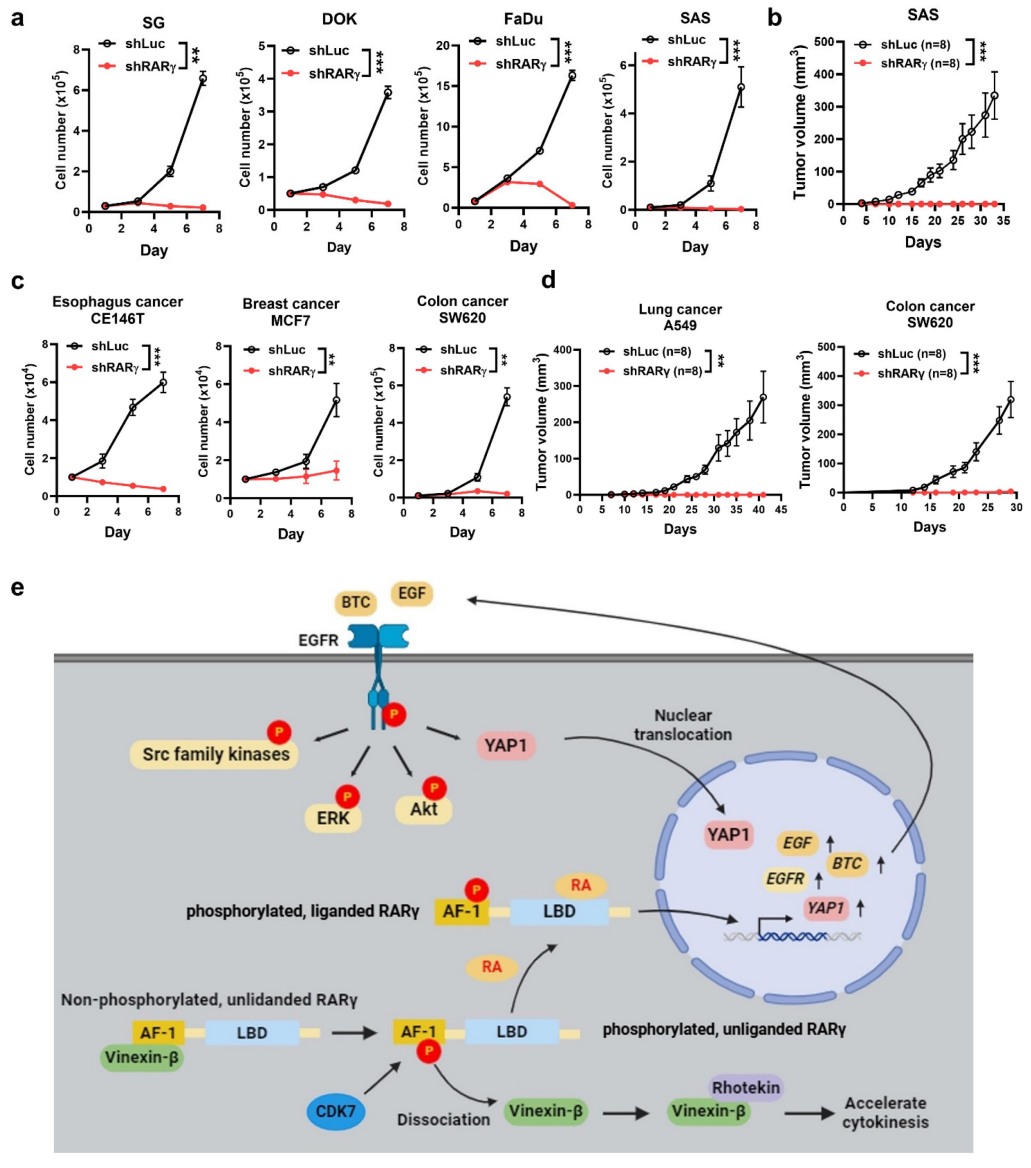
** Knockdown of RARγ abolished cell growth in HNC and various types of human cancers. (a)** Knockdown of RAR**γ** with shRNA suppressed the proliferation of SG, DOK, FaDu, and SAS cells. **(b)** Knockdown of RAR**γ** abolished tumorigenicity in a xenografted nude mouse model. Mice were inoculated subcutaneously with RAR**γ**-knockdown (shRAR**γ**) or control (shLuc) cells. Tumor volumes were measured twice a week. **(c)** Knockdown of RAR**γ** significantly suppressed the proliferation of esophageal (CE146T), breast (MCF7), and colon (SW620) cancer cells. **(d)** Knockdown of RAR**γ** abolished tumor growth in nude mice xenografted with lung (A549) and colon (SW620) cancer cells (*p < 0.05; **p < 0.01; ***p < 0.001). **(e)** A graphic summary of RAR**γ**-mediated growth promotion in HNC.

The growth-promoting effect of oncogenic RARγ isoforms prompted us to investigate whether an RARγ-targeting approach could serve as a viable therapeutic or chemopreventive strategy for HNC. To explore this, we tested whether knocking down RARγ isoforms using specific shRNAs could suppress the growth-promoting effect in HNC and various cancer cells. Representative RT-qPCR results demonstrated the mRNA levels of RARγ in FaDu and SAS cells transduced with lentiviral vectors expressing shRNA specific to RARγ1, 2, 4 (Supplemental Fig. S5). Figure 7a demonstrated that shRNA-mediated knockdown of RARγ completely blocked the proliferation of immortalized normal oral keratinocyte (SG), dysplastic oral keratinocytes (DOK), and HNC cells (FaDu and SAS) *in vitro*. Knockdown of RARγ similarly abolished tumor formation in nude mice xenografted with SAS cells (Fig. 7b), implying the clinical potential of RARγ-targeting approach in HNC. We next examined whether such an RARγ-targeting approach could also be a valid strategy for the treatment of various human cancers other than HNC. Knockdown of RARγ induced remarkable growth inhibition in esophageal (CE146T), breast (MCF7), and colon (SW620) cancer cells (Fig. 7c), and abolished tumor formation in nude mice xenografted with lung (A549) and colon (SW620) cancer cells (Fig. 7d). The above results indicated that RARγ-targeting could be a promising approach for the treatment of human cancers.

## Discussion

There are several unique findings in our study. First, we provide a comprehensive overview regarding the expression of RARγ isoforms in human cancers. The presence of multiple RARγ isoforms in each tumor indicates that the biological functions of RARγ should be interpreted as a collective effect of these isoforms, rather than a single RARγ molecule. Second, we uncover a previously unknown mechanism of cell cycle regulation involving the interaction between RARγ and vinexin-β. Third, we provide a solid link between RARγ and EGFR signaling pathways, demonstrating that the RARγ1, 2, 4-mediated promotion of HNC growth is primarily driven by autocrine, ligand-dependent activation of EGFR, alongside the coordinated activation of downstream signaling pathways. Finally, through both *in vitro* and *in vivo* models, we demonstrate that RARγ-targeting strategy could be a promising therapeutic and chemopreventive approach for both HNC and various kinds of human cancers. A graphic summary regarding the mechanisms of oncogenic RARγ isoform-enhanced head and neck malignancy is provided in Fig. 7e.

In this study, we demonstrated that RARγ1, 2, 4 activate EGFR and downstream signaling pathways. Although it was shown that EGFR activation could also be induced by EGFR overexpression in a ligand-independent manner 25, our results indicate that, in HNC cells, RARγ-mediated EGFR activation is ligand-dependent (Fig. 5e and 5f). We additionally demonstrated that both EGFR (Fig. 6b) and its high-affinity ligands could both be up-regulated by RARγ isoforms (Fig. 6d and Supplementary Fig. S4), implying that the RARγ-mediated growth-promotion of HNC is mainly dependent on EGFR activation in an autocrine manner. In support of our findings, a previous study also found that low-dose RA treatment increased EGF secretion in cultured HNC cells 27. Another plausible mechanism for RARγ-mediated EGFR activation may involve the interaction between RARγ and vinexin-β. It is shown that EGF enhances the binding of vinexin-β to the E3-ubiquitin ligase c-Cbl, which subsequently delays endocytosis and degradation of activated EGFR 28. Whether such regulatory mechanism additionally contributes to RARγ-mediated EGFR activation requires further investigation.

In the activation model of RARα, Ser^369^ phosphorylation (Ser^299^ of RARγ4) at the LBD by p38MAPK/MSK1 is essential for docking of transcription factor IIH (TFIIH) to form a RARα-TFIIH complex. The CDK7 subunit of TFIIH subsequently phosphorylates Ser^77^ (corresponding to Ser^7^ of RARγ4) at the AF-1 domain of RARα, allowing the RARα-TFIIH complex to target the RA response elements located in the promoter of responsive genes 21. However, we demonstrated that the phospho-defective mutant of RARγ4 (RARγ4-S299A) did not affect Ser^7^ phosphorylation and vinexin-β association (Fig. 3e), indicating that Ser^299^ of RARγ may have a regulatory role different from Ser^369^ of RARα. Since the phosphorylation status of Ser^299^ (S299A or S299E) of RARγ4 possess minimal effect on both cell growth (Fig. 2e) or EGFR activation/phosphorylation (Fig. 5b), the functional role of Ser^299^ in RARγ4 was not further addressed in this study. In addition, we noticed that phosphorylation of Ser^299^ is apparently required for RARγ-mediated Src activation (Fig. 5b). Thus, it is likely that RARγ4-mediated Src activation may be involved in the modulation of cellular functions other than proliferation. Supporting this notion, a previous study demonstrated that suppression of c-Src signaling inhibited RARγ-mediated neuritogenic differentiation in neuroblastoma cells 29.

We demonstrated that RARγ knockdown abolished proliferation of immortalized oral keratinocytes (SG), dysplastic oral keratinocytes (DOK) and HNC cells (Fig. 7a), and prevented *in vivo* growth of HNC (Fig. 7b). Since RARγ isoform dysregulation occurs early in carcinogenesis of HNC (Fig. 1), RARγ-targeting approach could be a promising approach for both therapeutic and chemopreventive usage of HNC. Similarly, targeting RARγ could also be an effective treatment in other human cancers, as demonstrated in our current findings (Fig. 7c and 7d**)** and results from previous studies 30-33. The feasibility of RARγ-targeting approach for cancer therapy is also supported by a recent study showing that RARγ may participate in driving the expression of stemness genes and promote self-renewal of colorectal cancer cells 34. In addition, RARγ (RARγ1) could also regulate a differentiation-apoptosis switch 35 and directly modulate DNA damage-induced, RIPK1-initiated apoptosis and necroptosis 17. Future explorations will be mandatory to reveal the role of RARγ isoforms in modulating stemness and/or cellular death machinery.

For RARγ-targeting, in addition to the increasingly popular RNA-based approaches 36, pharmacological antagonism of RARγ is another appealing strategy to achieve RARγ-targeting cancer therapy. Currently, there are several selective RARγ antagonists available, including AGN205728, MM11253, and LYS2955303 37. AGN205728 was shown to induce cell cycle arrest and caspase-independent apoptosis in prostate cancer cells 32. Notably, AGN205728 also inhibited the colony-forming ability of cancer stem cell (CSC)-like cells in a low nanomolar (nM) concentration, and synergistically enhanced the cytotoxic effects of several chemotherapeutic agents 32. Although these compounds are still under development as final drugs, the anti-tumor effects observed from AGN205728 further strengthen the idea that oncogenic RARγ-targeting could be an effective treatment for various types of human cancers.

In summary, our study provides novel mechanistic insights for RARγ isoform-mediated growth-modulation of HNC. RARγ-targeting approach could be a promising therapeutic and chemopreventive strategy for treatment of HNC and other types of human cancers.

## Methods

### Human tissues

Twenty pairs of fresh-frozen oral squamous cell carcinoma (OSCC) tissues with their corresponding adjacent non-tumor epithelia were used to study the expression patterns of RARγ isoforms in the RT-PCR experiments. Formalin-fixed, paraffin-embedded tissues from normal oropharyngeal epithelia (n=10), non-tumor epithelia adjacent to OSCC (n=10) and OSCCs (n=10) were obtained for immunohistochemical study to reveal the expression patterns of RARγ isoforms.

### Cells and reagents

Human primary oral keratinocytes (HOK) were purchased from ScienCell and were cultured in OKM (ScienCell). Human dysplastic oral keratinocyte cell line (DOK) and various HNC cell lines (FaDu, HSC3, OECM1, OC3, SAS) were routinely maintained as previously described 36. Human immortalized oral keratinocytes (SG), human esophageal (CE146T), lung (A549), breast (MCF-7), and colon (SW620) cancer cell lines were cultured in DMEM medium supplemented with 10% fetal bovine serum (FBS). All cells were incubated in 5% CO_2_ at a 37°C incubator. Erbitux (Cetuximab) and Erlotinib were purchased from Selleck Chemicals.

### RT-PCR and qRT-PCR

Total RNA was extracted using REzol C&T RNA extraction reagent (Protech Technology). One microgram (μg) of total RNA was reverse-transcribed into complementary DNA (cDNA) by an MMLV reverse transcription kit (Promega). RT-PCR was performed using RAR isoform-specific primers (Supplementary Table S2) in a PCR System (Applied Biosystems). Quantitative RT-PCR (qRT-PCR) was performed using EGFR- or YAP1-specific primers (Supplemental S2) and the qPCRBIO SyGreen Mix (PCRBiosystems) reacted in StepOne Real-Time PCR System (Applied Biosystems). Relative expression levels of EGFR and YAP1 were calculated according to the 2^-ΔΔCt^ method.

### Construction of expression vectors of various RARγ isoforms and RARγ isoform mutants

Plasmids pCMV6-RARγ1, pCMV6-RARγ2, and control vector (pCMV6) were purchased from Origene Technologies. Coding regions of human RARγ4 and RARγ5 were obtained by RT-PCR amplification using appropriate linker primers with AsiAI and BstBI restriction sites at its 5' and 3' ends (Supplementary Table S3). PCR fragments were then digested and cloned into pCMV6, resulting in pCMV6-RARγ4 or pCMV6-RARγ5 constructs. Subsequently, the pCMV-RARγ1, 2, 4, 5 plasmids were individually digested with EcoRI and PmeI. The resulting fragments of RARγ isoforms were then separately ligated into EcoRI/PmeI sites of pLVX-EF1α-IRES-ZsGreen1 (Takara) to generate pLVX-EF1α-RARγ isoform-IRES-ZsGreen1. The RARγ4 mutants were generated by site-directed mutagenesis with primers designed using NEBuilder (New England Biolabs) (Supplementary Table S4) The DNA fragments of the RARγ4 mutants were subcloned into SmaI/BstBI sites to construct the pLVX-EF1α-RARγ isoform-IRES-ZsGreen1 plasmids.

### Lentivirus production

Lentiviral vectors expressing human RARγ-specific short hairpin RNAs (TRCN0000021232; target sequence: 5'-CAATGACAAGTCCTCTGGCTA-3') were obtained from the National RNAi Core Facility, Academia Sinica, Taiwan. For lentivirus production, 293T cells were transiently transfected with either pLVX-EF1α-RARγ-IRES-ZsGreen1 or pLKO.1-shRARγ, along with the packaging constructs pSPAX2 and the VSV-G expression plasmid pMD2G, which were kindly provided by Didier Trono (Addgene plasmids #12260 and #12259). The media containing lentiviral particles were harvested 48 hours after transfection.

### Animal experiments

All animal experiments were approved and monitored by the Institutional Animal Care and Use Committee of the National Cheng Kung University (#110061). A total of 3 × 10^5^ cells were suspended in phosphate-buffered saline (PBS) and subcutaneously inoculated into eight-week-old NOD/SCID mice. Tumor volume was measured twice a week and calculated as length × width^2^ × 1/2.

### Co-immunoprecipitation

Cells were harvested and lysed with lysis buffer (20 mM Tris-HCl (pH 7.5), 150 mM NaCl, 1 mM Na_2_EDTA, 1 mM EGTA, 0.2% Triton, and 1 mM PMSF). Cell lysates were incubated with anti-Flag (Sigma) or anti-vinexin (Abnova) antibodies at 4℃ overnight. The immunocomplexes were precipitated by protein A/G-agarose (Santa Cruz) and then subjected to SDS-PAGE for immunostaining analysis.

### Immunoblotting

Cell lysates (15 μg/lane) were separated by SDS-PAGE and transferred to a PVDF membrane (Amersham). Then, the blots were developed using specific primary antibodies (Supplemental Table S5). Horseradish peroxidase-conjugated secondary antibodies (Jackson) were used for signal development to locate the primary antibodies by the chemiluminescence method.

### Time-lapse microscopy

Cells were placed on a preheated stage at 37℃ in 5% CO_2_. Cell images were recorded every 15 min for 24 hr using a Nikon TE2000-E microscope. Rounded replicating cells before furrow formation were considered as the starting time point of abscission. The duration of abscission was calculated from the starting time until the completion of cytokinesis.

### Kinase array

The phosphorylation status of kinases in SAS cells was detected using the Human Phospho-Kinase Array Kit (R&D Systems). Whole-cell lysates (200 μg) were incubated with antibody array membranes at 4℃ overnight. Subsequently, the membranes were incubated with detection antibodies and then treated with streptavidin-horseradish peroxidase. The signals were then developed and detected using the chemiluminescence method. Signal intensities were quantified with ImageJ software.

### Confocal microscopic analysis

Cells were fixed with 4% formaldehyde and permeabilized with 0.2% Triton X-100. After blocking, cells were incubated with primary antibodies against RARγ, RARγ1, RARγ2, flag, EGFR, or YAP1 (Supplemental Table 5). Alexa Fluor 488- or Alexa Fluor 594-conjugated goat anti-mouse or goat anti-rabbit IgG were used as secondary antibodies (Jackson ImmunoResearch). Cells were then stained with DAPI (Sigma) to visualize the nuclei. Specific fluorescence signals were detected using Olympus FV3000 Confocal Laser Scanning Microscope.

### Immunohistochemistry (IHC)

Paraffin-embedded tissue sections of 4-μm thickness were prepared. After deparaffinization and rehydration, tissue sections were blocked with Peroxidazed 1 (Biocare), followed by incubation with primary antibodies against RARγ1 or RARγ2 (Supplemental Table 5) at 4°C overnight. Tissue sections were then incubated with horseradish peroxidase (HRP)-conjugated secondary antibodies at room temperature for 2 h. Specific staining signals were then developed with 3,3'Diaminobenzidine (Santa Cruz). The slides were then counterstained with hematoxylin, and observed under a light microscope.

## Supplementary Material

Supplementary figures and tables.

## Abbreviations

RAR: retinoic acid receptor
HNC: head and neck cancer
OSCC: oral squamous cell carcinoma
EGF: epidermal growth factor
YAP: yes-associated protein
BTC: betacellulin
TGFA: transforming growth factor α
HB-EGF: heparin-binding epidermal growth factor
CDK: cyclin-dependent kinase
