# Supplementary material for: Oncogenic RARγ isoforms promote head and neck cancer proliferation through vinexin-β-mediated cell cycle acceleration and autocrine activation of EGFR signal
Source: Int J Biol Sci. 2025 Jan 1;21(1):1–16. doi: 10.7150/ijbs.100351 (PMC11667820; doi:10.7150/ijbs.100351)
Supplement: Supplementary file 1 — Supplementary figures and tables. [file ijbsv21p0001s1.pdf]

Figure S1. Confirmation of RARγ isoforms expression in lentiviral transduced cells. (a, b) Western blot results of exogenous RARγ were detected in ~~FaDu and~~ SAS cells expressing either (a) RARγ4 and its mutants or (b) RARγ1, 2, 4, 5 isoforms.

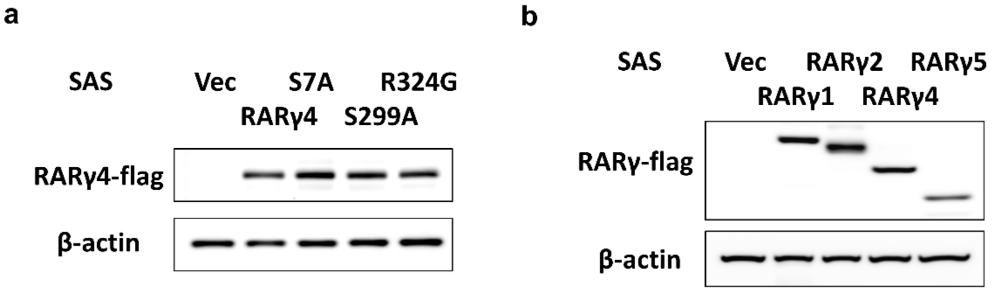

Figure S2. Transcriptional levels of *EGFR* and *YAP1* were up-regulated by RARγ1, 2, 4, but not RARγ5 in FaDu cells. Expression of *EGFR* and *YAP1* were detected in FaDu cells expressing RARγ1, 2, 4, 5 (*, $p < 0.05$; **, $p < 0.01$).

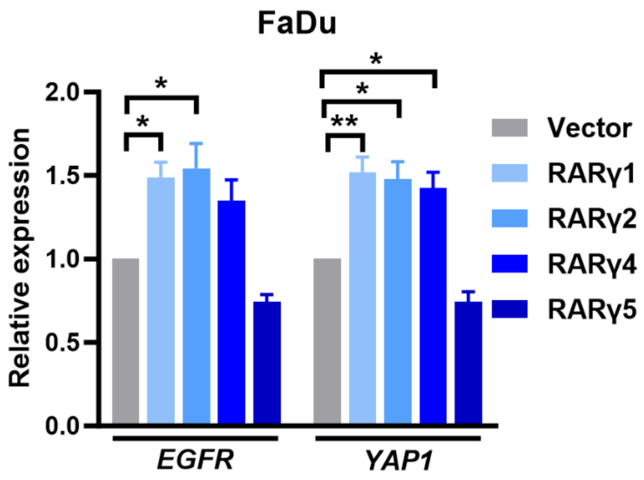

Figure S3. Inhibition of EGFR signaling suppressed RARγ1, 2, 4-mediated growth-promotion in FaDu cells. FaDu cells expressing RARγ1, 2, 4 were subjected to treatment with EGFR inhibitors (Erlotinib or Erbitux) at the specified concentrations. Cell numbers were determined for each group on day 7. (#, vector vs RARγ1, 2. *, RARγ1, 2, 4 vs EGFR inhibitor treated group. #, $p < 0.05$; ## or **, $p < 0.01$; ***, $p < 0.001$.)

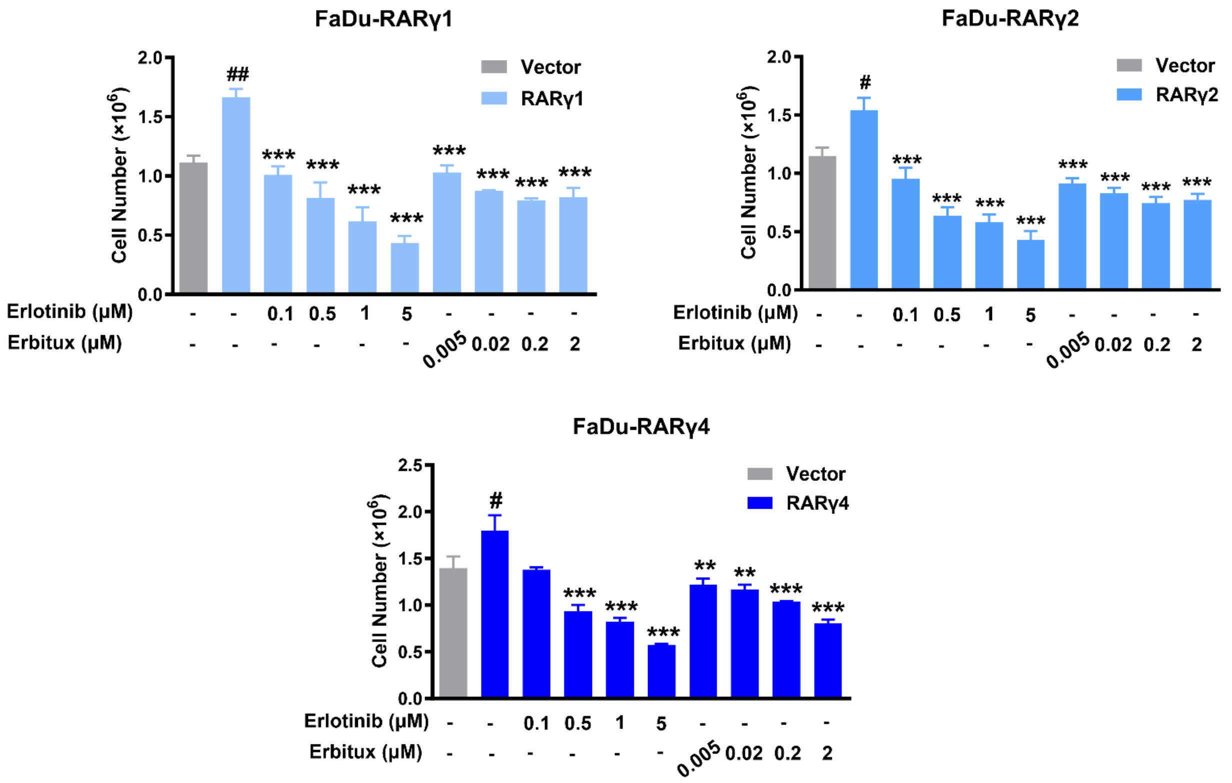

Figure S4. EGFR ligands BTC and EGF might contribute to RARγ-mediated EGFR activation. Expression of EGFR ligands *BTC*, *EGF*, *HB-EGF*, and *TGFA* were detected in FaDu cells expressing RARγ1, 2, 4, 5 (*, $p < 0.05$; **, $p < 0.01$; ***, $p < 0.001$).

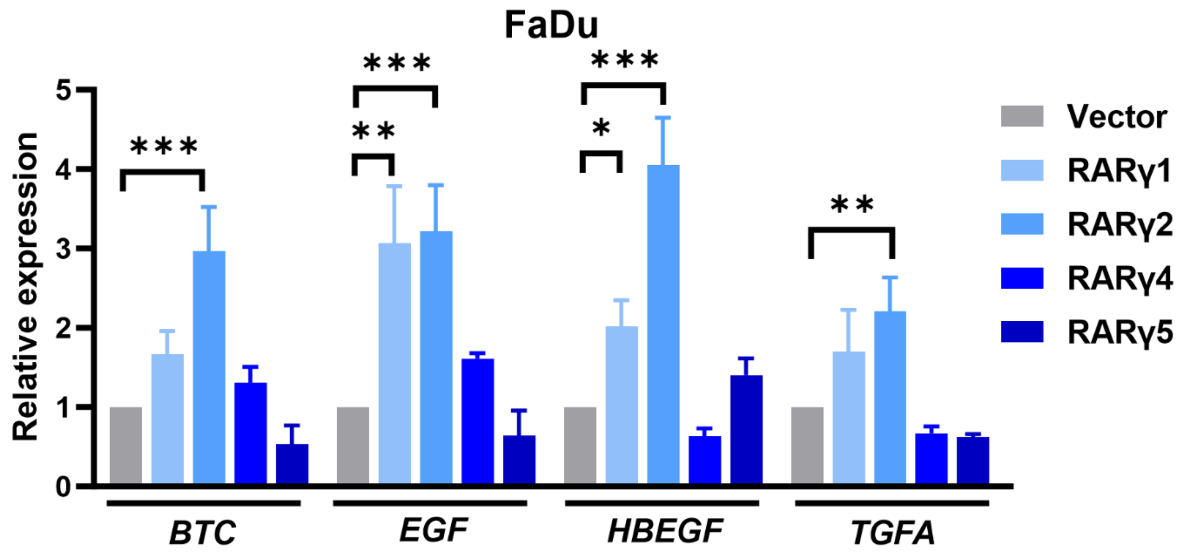

Figure S5. Knockdown efficiency of shRARγ in FaDu and SAS cells. Detection of RARγ expression in FaDu and SAS cells transduced with lentiviral vectors expressing shRNA specific to RARγ1, 2, 4.

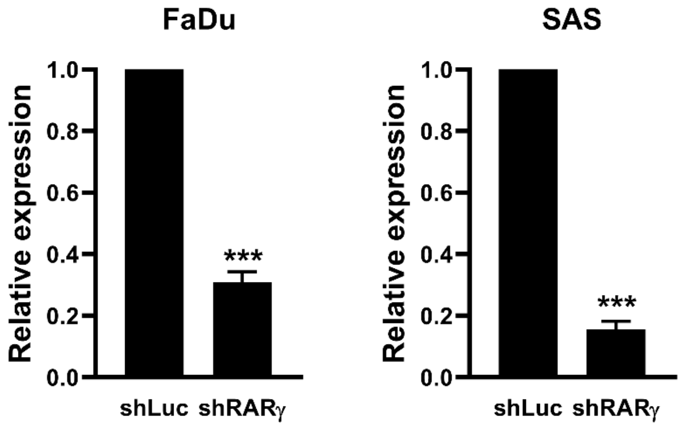

Table S1. Changes of kinase phosphorylation levels in SAS cells expressing either RARγ4 or RARγ4-R324G (compared to vector control)

Target	RARγ4 / Vector	*p-value*	R324G / Vector	*p-value*
LCK^{Y394}	1.4766	0.0269	1.0702	0.4483
HSP60	1.427	0.0014	1.2417	0.0036
$CREB^{S133}$	1.4081	0.0264	1.0735	0.3916
$HSP27^{S78/S82}$	1.3158	0.0001	1.1046	0.0101
$EGFR^{Y1086}$	1.2777	0.0088	0.8525	0.0547
GSK-3β^{S9}	1.2294	0.0105	1.0337	0.0133
$STAT3^{S727}$	1.1979	0.0525	0.8835	0.1105
STAT5a/$b^{Y694/Y699}$	1.1737	0.0119	1.0512	0.0581
Lyn^{Y397}	1.1146	0.1659	1.0015	0.9940
GSK-3α/$\beta^{S21/S9}$	1.1087	0.1603	0.9502	0.2555
Yes^{Y426}	1.1292	0.0156	0.8203	0.0362
$STAT1^{Y701}$	1.0895	0.409	0.9639	0.0413
MSK1/$2^{S376/S360}$	1.0848	0.3568	0.8791	0.0481
$WNK1^{T60}$	1.0635	0.0191	0.9304	0.0184
$PYK2^{Y402}$	1.0556	0.0475	0.9671	0.0371
β-catenin	1.053	0.1298	0.9838	0.6275
Akt1/2/3^{S473}	1.0398	0.4197	1.0043	0.9335
$PRAS40^{T246}$	1.0315	0.5524	0.9281	0.3391
$eNOS^{S1177}$	1.0157	0.9042	0.6614	0.1427
$STAT6^{Y641}$	1.027	0.862	1.0129	0.8707
Src^{Y419}	1.0169	0.4815	0.9474	0.0047
RSK1/$2^{S221/S227}$	0.9968	0.9633	0.9164	0.0552
JNK1/2/$3^{T183/Y185,T221/Y223}$	0.9744	0.6956	1.0669	0.2734
$STAT2^{Y689}$	0.9704	0.0949	0.9730	0.2242
PLC-γ^{Y783}	0.9613	0.4316	0.9039	0.0708
$p53^{S392}$	0.9527	0.0878	0.8955	0.1192
Akt1/2/3^{T308}	0.9501	0.3995	0.8014	0.0444
RSK1/2/$3^{S380/S386/S377}$	0.9373	0.1799	0.7954	0.0297
$p70S6K^{T389}$	0.9282	0.3215	0.8783	0.1862
ERK1/$2^{T202/Y204, T185/Y187}$	0.9276	0.1606	0.7313	0.0133
P38$\alpha^{T180/Y182}$	0.9273	0.0927	0.9536	0.1672
Fgr^{Y412}	0.924	0.2048	0.9051	0.5508
PDGFRβ^{Y751}	0.9098	0.2735	0.8915	0.1282
c-Jun^{S63}	0.8952	0.1668	0.5616	0.0082

Chk-2^{T68}	0.8951	0.1556	0.7990	0.0618
STAT3^{Y705}	0.8779	0.3042	0.8922	0.3516
p53^{S46}	0.8675	0.0241	0.9445	0.2101
p53^{S15}	0.7349	0.0124	0.7067	0.0808
p70S6K$^{T421/S424}$	0.6212	0.0127	0.6534	0.0859

Table S2. Primers used for RT-PCR and qRT-PCR

Name	Forward primer (5’ to 3’)	Reverse primer (5’ to 3’)	Tm
	**For RT-PCR**		
RARγ1,4,5	GCGGTTTGGGAGAAAATGTGTC	GTTCTTCTGGATGCTTCGGCG	59℃
RARγ2,3	CGCCGGACTTGAATCTTTT	CTGGCAGAGCGAGGGGAAAGT	60℃
β-actin	TCCACCTTCCAGCAGATGTG	CAACTAAGTCATAGTCCGCCTAGAA	60℃
	**For qRT-PCR**		
EGFR	CAGCGCTACCTTGTCATTCA	TGCACTCAGAGAGCTCAGGA	60℃
YAP1	TCTTCAACGCCGTCATGAAC	GGTCAGTGTCCC AGGAGAAA	60℃
BTC	CCTCTTCGGAAACGTCGTAAAAG	CTTGCCACCAACCTGGAGGTAA	60℃
EGF	CCAGCTCTGCGTTCCTCTTA	GCAAACAGCAAAAATGGTTGTGG	60℃
HB-EGF	TGTATCCACGGACCAGCTGCTA	TGCTCCTCCTTGTTTGGTGTGG	60℃
TGFA	GGTCCGAAAACACTGTGAGTGG	CAAACTCCTCCTCTGGGCTCTT	60℃
β-actin	TCCACCTTCCAGCAGATGTG	CAACTAAGTCATAGTCCGCCTAGAA	60℃

Tm: annealing temperature

Table S3. Linker primers for RARγ4 and RARγ5 with AsiAI and BstBI restriction enzyme sites

Name	Forward primer (5' to 3')	Reverse primer (5' to 3')	Tm
RARγ4	TTTTGCGATCGCCATGGTGCCCAGCTCGCCCTC	CTTCACCTCTTTCTTCTTCTTGTTCC	55℃
RARγ5	TTTTGCGATCGCCATGGTGTACACGTGTCACCGC	CTTCACCTCTTTCTTCTTCTTGTTCC	55℃

Tm: annealing temperature

Table S4. Primers used for site-directed mutagenesis of RARγ4

Name	Forward primer (5' to 3')	Reverse primer (5' to 3')	Tm
RARγ4-S5A	TGCCCAGCGCGCCCTCGCCCCCT	AGGGGGCGAGGGCGCGCTGGGCA	55℃
RARγ4-S7A	TGCCCAGCTCGCCCGCGCCCCCT	AGGGGGCGCGGGCGAGCTGGGCA	55℃
RARγ4-S7E	TGCCCAGCTCGCCCGAGCCCCCT	AGGGGGCTCGGGCGAGCTGGGCA	55℃
RARγ4-S299A	CCGGCGGCCCGCCCAGCCCTACA	TGTAGGGCTGGGCGGGCCGCCGG	55℃
RARγ4-S299E	CCGGCGGCCGAGCAGCCCTACA	TGTAGGGCTGCTCGGCCGCCGG	55℃
RARγ4-R324G	GGGAGCTGAAGGGGCCATTACTC	GAGTAATGGCCCTTTCACTCCC	55℃

Tm: annealing temperature

Table S5. Primary antibodies used in this study

Antibodies	Manufacturers	Catalog	Applications					
Akt	Cell Signaling Technology	2920	WB	1:2000				
pAkt^{S473}	Cell Signaling Technology	9271	WB	1:1000				
All RARγs	Santa Cruz Biotechnology	sc-398065			IF	1:100		
β-actin	Abcam	ab6276	WB	1:10000				
EGFR	Santa Cruz Biotechnology	sc-03	WB	1:3000	IF	1:500		
pEGFR^{Y1068}	Abcam	ab40815	WB	1:1000				
pEGFR^{Y1086}	R&D	MAB8967	WB	1:1000				
ERK	Cell Signaling Technology	9102	WB	1:3000				
pERK$^{T202/T204}$	Cell Signaling Technology	9101	WB	1:1000				
Flag	Sigma	F1804	WB	1:1000	IF	1:300	IP	1:100
RARγ1	Cell Signaling Technology	8965	WB	1:1000				
RARγ1	Sigma	HPA053883			IF	1:100	IHC	1:50
RARγ2	Abcam	ab51368	WB	1:200	IF	1:100	IHC	1:50
Rhotekin	Abnova	H00006242-M01	WB	1:2000				
pSrc^{Y416}	Cell Signaling Technology	2101	WB	1:1000				
Vinexin	Abnova	H00010174-B01P	WB	1:1000			IP	1:100
YAP1	Abnova	H00010413-M01	WB	1:2000	IF	1:100		
